# Enhancing carbon-negative emission technologies through biomass integration

**DOI:** 10.1016/j.xinn.2025.101079

**Published:** 2025-08-08

**Authors:** Shijie Yu, Qinghai Li, Yanguo Zhang, Jinyue Yan, Hui Zhou

**Affiliations:** 1 Key Laboratory for Thermal Science and Power Engineering of Ministry of Education, Beijing Key Laboratory of CO_2_ Utilization and Reduction Technology, Department of Energy and Power Engineering, Tsinghua University, Beijing 100084, China; 2 Department of Chemical and Biomolecular Engineering, National University of Singapore, Singapore 117585, Singapore; 3 Shanxi Research Institute for Clean Energy, Tsinghua University, Taiyuan, Shanxi 030000, China; 4 Department of Building Environment and Energy Engineering, The Hong Kong Polytechnic University, Hong Kong SAR 999077, China; 5 International Centre of Urban Energy Nexus, The Hong Kong Polytechnic University, Hong Kong SAR 999077, China

**Keywords:** carbon-negative emissions, bio-energy with carbon capture and storage, BECCS, carbon dioxide fixation, negative emission technologies, renewable energy, climate mitigation

## Abstract

Conventional biomass conversion technologies, such as combustion, gasification, and anaerobic digestion, are considered carbon neutral since the carbon released originates from the atmospheric CO_2_ absorbed during biomass photosynthesis from the perspective of principles. By integrating carbon capture and storage (CCS) with bio-energy processes, the overall system can achieve a carbon-negative footprint. Various CCS technologies can be employed depending on the applicability and efficiency, which vary according to the CO_2_ parameters. The integration of bio-energy with carbon capture and storage (BECCS) encompasses technologies such as fermentation, oxy-fuel combustion, chemical looping, calcium looping, and alkaline thermal treatment with carbon mineralization. These methods exhibit substantial potentials, especially when the released CO_2_ is concentrated or readily available for storage, leading to carbon-negative emission. Moreover, carbonization technologies such as pyrolysis and hydrothermal carbonization convert biomass carbon into solid materials, rendering them carbon negative in principle of carbon flow. This comprehensive review paper explores a wide range of biomass-based carbon-negative emission technologies, in contrast to previous reviews that typically focus on a specific pathway or technology. It systematically compares these technologies in terms of CO_2_-related parameters, energy conversion efficiency, carbon negativity, economic viability, and commercialization status. Moreover, the review delves into the challenges and opportunities inherent in advancing carbon-negative emission technologies driven by biomass, offering valuable insights for future developments in this critical field.

## Public summary

• Carbon-negative emission technologies can be achieved through biomass integration.
• Biomass conversion with CCS and integrated BECCS technologies are categorized.
• Carbonization technologies convert biomass carbon into solid materials with carbon-negative effect.
• Different carbon-negative emission technologies enabled by biomass are compared.

## Graphical abstract

**Figure:**
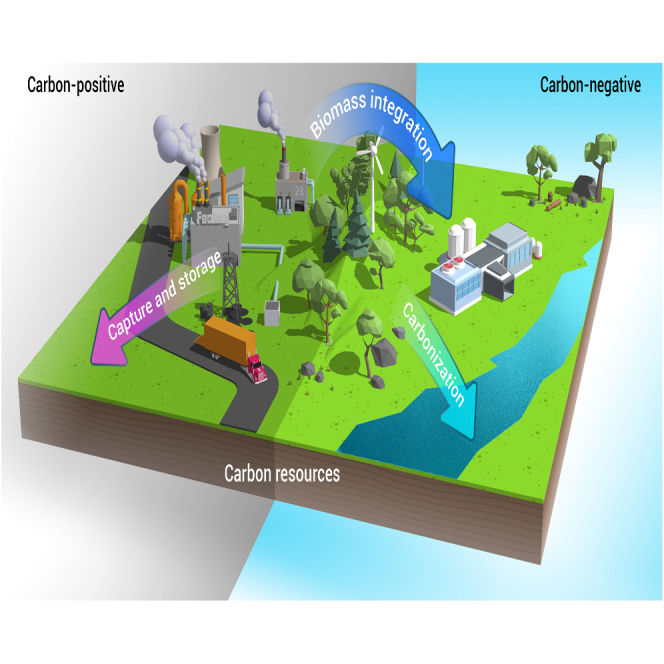

## Introduction

Global atmospheric CO_2_ concentrations have increased by around 100 ppmv over the past two centuries due to anthropogenic CO_2_ generated by agricultural and industrial activities.^1^^,^^2^^,^^3^ At the Paris Climate Conference (COP21) in December 2015, an agreement was negotiated by 195 countries, which set the goal to hold the global average temperature increase to less than 1.5°C above pre-industrial levels.^4^^,^^5^ The 1.5°C limitation may require the CO_2_ concentration not exceeding 450 ppmv,^6^ a much harder target compared with the 550 ppmv plan proposed in the IPCC 4th Assessment Report from 2007.^7^ To meet the CO_2_ goal will require not only zero emissions, but also negative CO_2_ emissions, the permanent removal of CO_2_ from the atmosphere, which is critical since it is the only way to bridge the gap between the high CO_2_ concentration and the desired target.^8^^,^^9^ In addition, certain sectors such as transportation fuels pose challenges in acquiring carbon capture, necessitating the implementation of carbon-negative strategies to offset these emissions.^10^^,^^11^ According to an updated model, negative emissions of 7–11 Gt carbon per year is needed in the worst case to meet the 2°C target. Even in the best case, negative emission of 0.5–3 Gt carbon per year is needed.^12^

**Figure 1:**
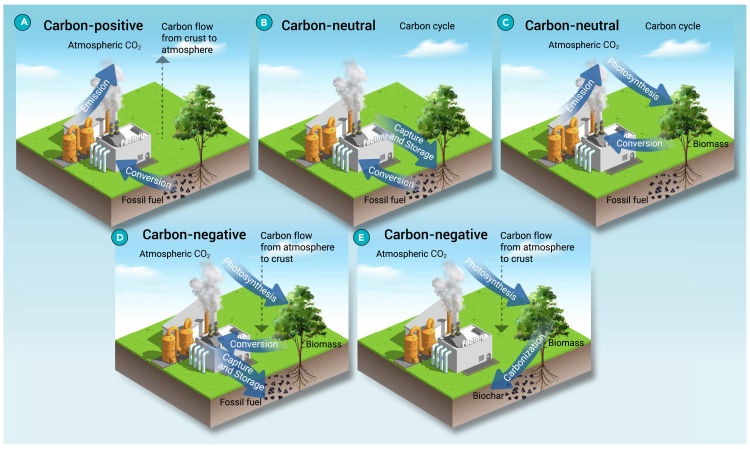
Schematics of ecological carbon substance flows for different technological scenarios, categorized as carbon positive, carbon neutral, and carbon negative (A) Carbon-positive scenario in which fossil fuel is converted as energy sources and the resulting CO_2_ is emitted directly. (B) Carbon-neutral scenario in which fossil fuel is converted as energy sources and the resulting CO_2_ is captured and stored. (C) Carbon-neutral scenario in which biomass is converted as energy sources and the resulting CO_2_ is emitted directly. (D) Carbon-negative scenario in which biomass is converted as energy sources and the resulting CO_2_ is captured and stored. (E) Carbon-negative scenario in which biomass is directly carbonized and converted to biochar.

To date, there are several methods proposed to be possible for the achievement of negative carbon emissions, including direct air capture associated with carbon storage and utilization,^13^^,^^14^^,^^15^ enhanced weathering,^16^^,^^17^ afforestation and reforestation (AR),^18^^,^^19^ carbonization of biomass,^20^^,^^21^^,^^22^^,^^23^ and bio-energy with carbon capture and storage (BECCS).^24^^,^^25^^,^^26^^,^^27^ As shown in Figure 1, traditional fossil fuel utilization, such as coal combustion and natural gas combustion for electricity, is carbon positive. When the released CO_2_ is captured and stored, the system is carbon neutral or zero emission. The utilization of bio-energy is also carbon neutral since the carbon in bio-energy is from CO_2_ in the atmosphere. When carbon capture and storage (CCS) is introduced into bio-energy utilization (i.e., BECCS), the system is carbon negative. The direct conversion of biomass to solid biochar through carbonization is also carbon negative.

Carbon-negative emission technologies enabled by biomass show obvious potential in carbon emission reduction. For example, BECCS has gained widespread attention since it was proposed by Möllersten and Yan in 2000, and is even considered climate change’s “savior” technology.^28^^,^^29^^,^^30^ According to the Organisation for Economic Co-operation and Development (OECD) Environmental Outlook to 2050 released at the 2011 UN Climate Change Conference, meeting lower CO_2_ concentrations “depends significantly on the use of BECCS.”^31^ Moreover, Gough and Upham analyzed the cost and feasibility of meeting global CO_2_ concentration target by global energy-economy models, and found that BECCS plays a critical role in decreasing atmospheric CO_2_ concentration.^32^ Compared with other negative emission technologies (NETs), BECCS is regarded as a “low-hanging fruit” and could be integrated into the current energy systems.^33^^,^^34^ From the prediction of NET scenarios, the technical potential of BECCS varied from 0.5 to 20 Gt per year of carbon equivalent (Ceq.) in 2050.^8^^,^^35^^,^^36^^,^^37^^,^^38^^,^^39^^,^^40^^,^^41^^,^^42^^,^^43^ Moreover, other carbon-negative emission technologies, such as biomass carbonization technologies, also show significant carbon reduction potential, by which 3.4–6.3 Gt Ceq. carbon removal is expected to be delivered.^44^

**Figure 2:**
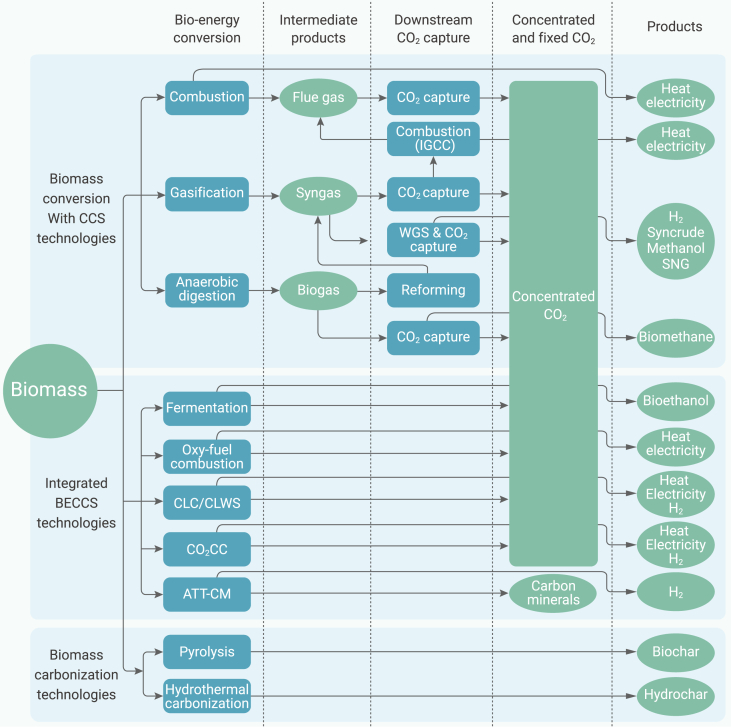
Different routes of carbon-negative emission technologies enabled by biomass, including biomass carbonization technologies, biomass conversion with CCS technologies, and integrated BECCS technologies

Based on the different conversion methods, bio-energy offers a variety of technologies to achieve CO_2_ negative effect.^45^ As shown in Figure 2, combustion, gasification, and anaerobic digestion can all be combined with CCS, while there are some integrated BECCS technologies, including biomass fermentation, oxy-fuel combustion (OFC), chemical looping, calcium looping, and alkaline thermal treatment with carbon mineralization (ATT-CM). Besides, pyrolysis and hydrothermal carbonization are typical carbonization technologies.

Although different carbon-negative emission technologies enabled by biomass are flourishing and there are many reviews summarizing the progress of each of these specific technologies, there is still no review that systematically summarizes and compares all carbon-negative emission technologies enabled by biomass. This review paper focuses on the carbon-negative emission technologies enabled by biomass, including carbonization technologies and the combination of bio-energy conversion technologies and carbon capture technologies. The produced CO_2_ parameters (temperature, concentration, and impurities), carbon negativity, energy efficiency, and technology status are compared. A detailed description of biomass utilization technologies and carbon capture technologies is beyond the scope of this article. An evaluation of full life cycle of carbon-negative emission technologies enabled by biomass and comparison with other NETs are also not included in this review.

## Biomass conversion with CCS technologies

### Combustion with CCS

Biomass can be combusted in a grate furnace, pulverized furnace, or fluidized bed furnace, with an adequate air supply.^46^^,^^47^^,^^48^ Similar to the combustion of coal, biomass combustion will produce some pollutants, such as CO, NO_x_, polycyclic aromatic hydrocarbons, and particulate matters (PMs).^49^^,^^50^ Since biomass usually contains more alkaline metals (especially potassium) than coal, during biomass combustion at ∼1,000°C the low-boiling point alkali metal compounds (mainly potassium chloride) is vaporized, causing fouling and corrosion of the boiler.^51^

It should be noted that, although biomass can be combusted alone, studies have shown that co-firing plant of biomass and coal is more efficient than dedicated biomass power plant.^52^^,^^53^ The co-firing ratio of biomass can vary from 5% to 60% (in energy terms), depending on different situations.^54^ For higher co-firing rates, the relatively low energy density of biomass may lead to efficiency penalty and increased costs. To avoid this limitation, indirect co-firing and parallel co-firing are proposed, where biomass is fed separately.^55^

The flue gas from combustion contains around 15% CO_2_, with the other gases being mainly N_2_, O_2_, and water vapor.^56^ Compared with carbon capture from a coal power plant, the capturing emissions from biomass power plants is anticipated to be similar despite biomass plants typically having smaller capacity.^57^^,^^58^ Compared with CCS of coal combustion, the advantage may be that low sulfur content in biomass results in lower SO_x_ in the flue gas, which is an impurity for the carbon capture process.^59^ The possible disadvantage is that the higher volume of flue gas due to high moisture content of biomass may lower the capture efficiency and increase the energy penalty. In addition, due to the low energy density of biomass feedstock, a circulating fluidized bed (CFB) is commonly used for biomass combustion.^60^ Capturing CO_2_ from CFB may result in higher cost since the high air input leads to lower CO_2_ concentration in the flue gas.

Numerous technologies exist for post-combustion carbon capture (PCC), including solvents, membranes, solid sorbents, and cryogenic methods, as shown in Table S1. The most common PCC technology is amine scrubbing, which can trap ∼90% CO_2_ from the flue gas. The regeneration of solvent is carried out at an elevated temperature of 100°C–140°C, where energy input is required.^61^ An economical and efficient pathway for post-combustion CO_2_ capture is still being explored.

### Gasification with CCS

Biomass gasification is the thermochemical conversion of biomass at 750°C–1150°C in the atmosphere of steam, CO_2_, or insufficient oxygen.^62^^,^^63^ Another idea of gasification is hydrothermal gasification, where biomass is converted in the water phase at 200°C–600°C under high pressure.^62^^,^^64^ The produced gas from gasification is called syngas, which is very versatile to be used for gas fuel, synthesis of transportation fuel (such as the Fischer-Tropsch process), and production of other chemical products (such as dimethyl ether). The composition of dry syngas can be various, but generally in the following range: H_2_ (15%–45%), CO (20%–60%), CH_4_ (1%–12%), CO_2_ (10%–40%), and N_2_ (0%–1%).^65^

**Figure 3:**
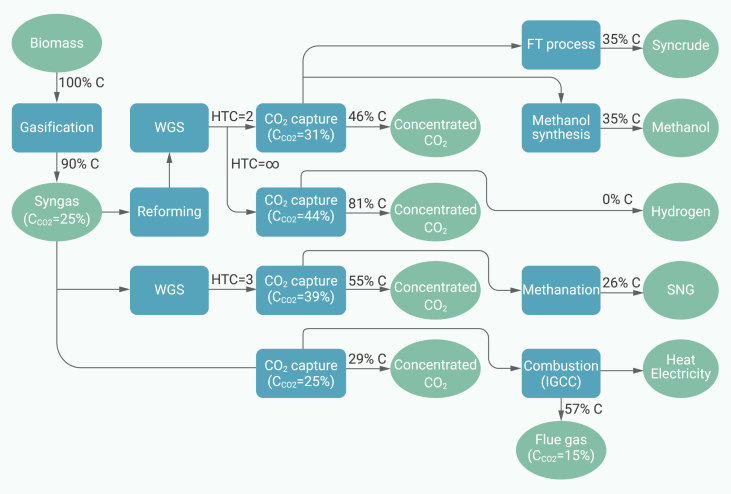
Main routes of gasification with CCS The assumptions and the calculation process can be found in Table S2.

Syngas can be used to generate electricity directly in a gas turbine, which is called biomass integrated gasification combined cycle (BIGCC),^66^ as shown in Figure 3. Without carbon capture, a net efficiency of 34% could be achieved; with carbon capture, the efficiency is 28%.^67^ Downstream fuel synthesis from syngas requires different H_2_-to-CO (HC) ratios, as shown in Figure 3. Methanol synthesis or Fischer-Tropsch (FT) synthesis require HC ratio to be 2,^68^ while methanation to synthetic natural gas requires HC ratio to be 3.^65^ For the proton exchange membrane fuel cells, H_2_ with CO concentration as low as 5–10 ppm can cause poisonous problems.^69^ The HC ratio can be adjusted by the water gas shift reaction (WGS), as shown in Reaction (1).

(Equation 1) $$\text{CO}+H_{2}O\;\to \;H_{2}+{\text{CO}}_{2}$$

After the WGS reaction, CO_2_ removal from syngas is necessary for downstream applications to increase the H_2_ and CO partial pressures and promote the conversion rate. Capturing CO_2_ from syngas has been commercially proven in other applications, such as the hydrogen production from fossil fuels for ammonia synthesis.^70^ The energy penalty for CO_2_ capture from syngas is theoretically lower than that of post-combustion capture, due to higher CO_2_ concentration in the syngas. From this perspective, gasification in air is less beneficial since N_2_ will be introduced to dilute the syngas.^71^

Similar to post-combustion capture, the CO_2_ capture with biomass gasification is usually based on the use of chemical or physical absorption (Table S1). Physical solvents are widely used when the CO_2_ concentration is high in the syngas. The advantage is that the energy for the regeneration is typically low, and only the pressurizing process is needed.

### Anaerobic digestion with CCS

Anaerobic digestion (AD) is a process that organic matter is degraded by microorganisms with the absence of air.^72^^,^^73^ Compared with the traditional one-stage AD process, the two-stage AD process, where the hydrolysis/acidogenesis and methanogenesis are split and separately optimized, have been reported to have a higher overall efficiency.^74^^,^^75^

The produced biogas usually contains 45%–70% CH_4_ and 25%–45% CO_2_, with a small amount of H_2_S (0.00001%–1%).^76^ Biogas could be upgraded to biomethane (97%–99% CH_4_ and 1%–3% CO_2_) with the CO_2_ separated and impurities removed.^77^ It should be noted that biogas could also be converted to syngas and/or hydrogen by reforming, then the carbon capture is similar to the process of syngas discussed above.^78^ A combination of oxy-reforming, WGS, and carbon capture processes was modeled, where high purity H_2_ could be produced with carbon-negative effect.^79^

The impurities in biogas include H_2_S, NH_3_, and siloxanes, which will poison the downstream carbon capture or biogas conversion processes, and generate pollutants (such as SO_x_ and NO_x_) if burned directly.^76^ Therefore, the purification of biogas is critical for its subsequent utilization.

CO_2_ is considered inert during biogas processes, while the separation of CO_2_ increases the concentration of methane and improves the quality of biogas. As shown in Table S1, the carbon capture from biogas is very similar to that from syngas. The common method is pressure swing adsorption with a molecular sieve, which has shown high selectivity.^80^ High-pressure water wash is another popular technology where CO_2_ and other impurities can be scrubbed by cascading water. The polymeric membrane is used commercially in the separation of CO_2_ from natural gas at high CO_2_ concentration, which could be also utilized in the separation in biogas.^81^ It should be noted there is no limitation on the application of post-combustion CO_2_ capture from biomethane combustion, which will increase the overall carbon-negative effect of AD-CCS.

## Integrated BECCS technologies

Besides the aforementioned BECCS technologies, CO_2_ could be captured or concentrated *in situ* during biomass utilization, that is, using the integrated BECCS technologies. The integrated BECCS technologies combine the processes of biomass utilization and carbon capture, which may reduce the capital and operational costs.

### Fermentation with carbon sequestration

The ethanol production from sugar is based on the fermentation of C6 sugars by enzymes.^82^^,^^83^ During the fermentation process, biomass can be converted into ethanol and CO_2_. The ethanol produced is usually called bioethanol. Bioethanol is the most common biofuel, accounting for more than 70% in the current global biofuel market. The first-generation bioethanol (1G bioethanol) is from sugar-based (such as sugar cane) or starch-based feedstock (such as corn).^84^ However, there are some concerns that the large-scale production of 1G bioethanol can cause competition with food, or with the land and water use for food supplies, which will affect global food security.^85^ The second-generation bioethanol (2G bioethanol) uses lignocellulosic biomass (such as wheat straw, maize stover, and grass) as the feedstock, with a pretreatment step to separate cellulose from hemicellulose and lignin.^86^^,^^87^ These biomasses can be grown in infertile land or produced together with food crops, which therefore can save valuable arable land.^88^

CO_2_ separation is part of the fermentation process, with very low influence on thermal efficiency. The CO_2_ concentration released from fermentation is around 85% in the gas product, while a 98.8%–99.6% concentration can be obtained after steam removal. The temperature of final CO_2_ stream is 25°C–50°C.^38^ Further treatment of CO_2_ is not necessary before compression and transport.

Water level below 250 ppm is suggested during CO_2_ transport to avoid corrosion, gas hydrate, and ice formation.^61^ The water vapor in CO_2_ streams is first cooled to below its dew point, and then glycol or solid adsorbent (e.g., molecular sieve) are employed for the dehydration.

After the fermentation, 60%–65% of carbon is left in the form of lignin and residue, 20%–30% carbon is converted into bioethanol, and 10%–15% carbon is in the form of CO_2_ and can be captured.^38^ It is estimated that approximately 765 g CO_2_ can be captured with the generation of 1 L bioethanol.^38^ The lignin portion of the biomass can also be used for power and heat generation, and thus the carbon negative effect will be improved greatly if post-combustion capture is added.^89^

### OFC

**Figure 4:**
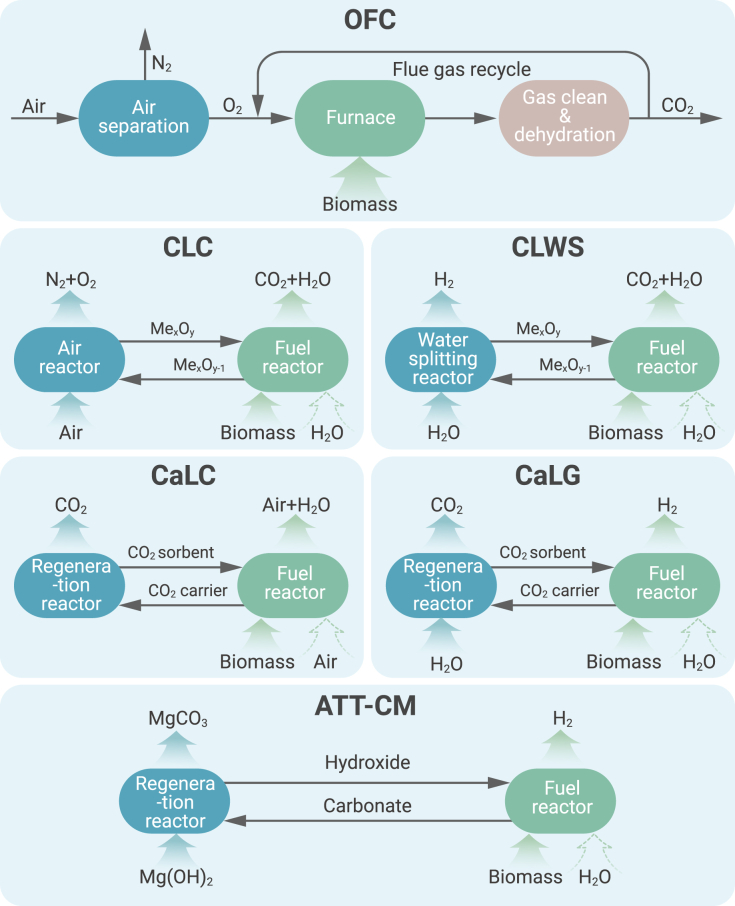
Principles of different integrated BECCS technologies, including OFC, CLC, chemical looping with water splitting (CLWS), calcium looping combustion (CaLC), calcium looping gasification (CaLG), and ATT-CM

OFC was proposed in the early 1980s to produce high purity CO_2_ stream for enhanced oil recovery (EOR).^90^ It is based on the idea of denitrification of air. The O_2_ in air can be separated by a cryogenic air method or membrane method and the combustion is in a relatively pure O_2_ stream. Recycled flue gas (RFG) is combined with oxygen to regulate the combustion temperature and prevent it from reaching excessive levels,^61^ as shown in Figure 4. The flue gas from OFC is mainly composed of CO_2_ and H_2_O, which can be separated easily. The carbon capture efficiency of an oxy-fuel power plant can be as high as 90%. It should be noted that the limitation of this technology is the energy penalties during air separation, which decreases the efficiency of the power plant.^91^^,^^92^

The flue gas contains mainly CO_2_ and water vapor, with some excess oxygen (∼3%). The net flue gas typically contains 70%–95% CO_2_, depending on the fuel input and combustion system.^91^ There may be some impurities such as SO_x_, NO_x_, HCl, and N_2_. Compared with combustion in air, the production of NO_x_ is decreased due to OFC, since NO_x_ is reduced by flame-generated hydrocarbons in the furnace by RFG, and N_2_ from the air is eliminated, which in turn reduces the amounts of thermal and promot-NO_x_.^61^ Currently, little information about particulates and volatile organic compounds is available. Co-firing of coal and biomass is tested in a 0.8 MW pilot-scale OFC fluidized bed. Up to 50% wood is used together with coal, and the flue gas has a CO_2_ concentration of 80%–90%.^93^ The technology of combustion in the mixture of oxygen and steam (hydroxy-fuel combustion) is also under investigation. In this technology, steam, instead of RFG is used to moderate the combustion temperature. This technology has an advantage of a reduced equipment size and the novel steam-gas turbomachinery is utilized. This study is at a very early stage, and further exploration is required.

### Chemical looping

The chemical looping process attempts to split the process of combustion/gasification into separated oxidation and reduction reactions using oxygen carriers (OCs) (usually metal oxides),^94^^,^^95^ as shown in Figure 4. Biomass could be *in situ* gasified by H_2_O or CO_2_, and combusted with the presence of OCs, which is also called *in situ* gasification-chemical looping combustion (iG-CLC).^96^ Another process, chemical looping oxygen uncoupling (CLOU) is also proposed, where molecular O_2_ was released from OCs, which could oxidize biomass instead of lattice oxygen to improve the poor solid/solid contact efficiency.^97^^,^^98^

The most advantageous part of chemical looping is that CO_2_ is not diluted by N_2_, where the concentration of CO_2_ varies from 60% to 98% as shown in Figure S1A,^96^^,^^99^^,^^100^^,^^101^^,^^102^ which is good for carbon sequestration and storage.^103^^,^^104^ According to different studies, the carbon capture efficiency of CLC varies from 75% to 100% at different temperatures from 700°C to 1,000°C, as shown in Figure S1B.^96^^,^^99^^,^^100^^,^^101^^,^^102^

More than 900 types of OCs have been tested in the laboratory and there are some reviews focusing on this aspect.^94^^,^^105^^,^^106^ The studies in recent years focus on dual metals, low-cost materials, and CLOU materials.^107^^,^^108^ Low-cost materials, such as iron ore, CaSO_4_/CaS, and industrial waste materials are suitable for biomass CLC. The low-cost mineral ilmenite works well, but further studies on reactor system and OC design are still needed to obtain a higher performance.^109^ The OCs are usually fluidized in the form of fine particles to increase the mass and heat transfer. Therefore, a critical problem is the stability of the OCs after multiple cycles. The reasons of deactivation may include agglomeration, attrition, carbon deposition, or sulfur poison. Similar to direct biomass combustion, biomass CLC also suffers from the issue of ash volatilization due to the high temperature in the fuel reactor. In addition to fouling and corrosion caused by ash volatilization, the interactions between ash and OCs may also cause the deactivation of OCs.^96^

Chemical looping with water splitting is a novel process where water is used to oxidize the OCs instead of air in CLC, as shown in Figure 4. In the fuel reactor, OCs are reduced by syngas produced from biomass gasification/reforming. OCs are oxidized by water in the water splitting reactor, and therefore, almost pure hydrogen could be produced from this process.^110^ FeO/Fe_3_O_4_/Fe_2_O_3_ are usually used as the OCs. The cycles could be FeO-Fe_3_O_4_ or FeO-Fe_3_O_4_-Fe_2_O_3_, depending on whether a third reactor is used to oxidize Fe_3_O_4_ into Fe_2_O_3_.^111^

### Calcium looping

CaO-based materials can absorb CO_2_ at high temperatures. Limestone and dolomite are commonly used as CaO precursors because they are inexpensive with a high CO_2_ capacity and fast kinetics.^112^ When *in situ* carbon capture is coupled with gasification/reforming, a hydrogen-rich gas could be produced, which has significant efficiency and economic improvements potentials. The ideal reaction could be described as Reaction (2)^113^

(Equation 2) $$C_{x}H_{y}O_{z}+H_{2}O+\text{CaO}\;\to \;H_{2}+{\text{CaCO}}_{3}$$

According to model simulations, with the addition of CaO, the H_2_ concentration could increase from ∼25% to more than 70% at 500°C, and the concentration of CO_2_ decreases from ∼20% to ∼0%.^65^ The experiments in a fluidized bed reactor also proved this result, where a gas with 71% H_2_ and almost no CO_2_ was produced using sawdust as the feedstock.^114^

The CaCO_3_ product can be transferred and calcined in a separate reactor at high temperatures (>800°C), with the release of the relatively pure CO_2_ stream. Therefore, the entire process could be also called calcium looping gasification (Figure 4).^115^ To obtain a pure CO_2_ stream from the calcination of CaCO_3_, the calcination cannot be carried out in air. Instead, a CO_2_-rich atmosphere is needed, which will increase the calcination temperature to 900°C–950°C due to the high CO_2_ partial pressure.^116^ Therefore, the high energy demand for the regeneration of CaO is a limitation of the calcium looping.^117^

The *in situ* carbon capture combustion of biomass with calcium looping, i.e., calcium looping combustion, is also proposed (Figure 4).^118^^,^^119^ During biomass combustion, CO_2_ is captured *in situ* by CaO, and thus the flue gas contains only N_2_, excess O_2_, and steam. Due to the thermodynamics of the carbon capture reaction, the combustion is limited to temperatures lower than 700°C. The 300 kW_th_ pilot test showed that the CO_2_ capture efficiencies varied between 70% and 95% using wood as the fuel.^120^ An economic and process analysis of this pathway has revealed this novel concept to be economically viable.^120^^,^^121^

Since the accumulation of a CaCO_3_ layer on the surface of CaO could limit the diffusion process, the conversion of CaO to CaCO_3_ is usually lower than 70% in the first cycle.^116^ After long-term tests, the carbonation extent decreases rapidly, which could be as low as less than 10% after 30 cycles. The deactivation may come from sintering, attrition, and reactions with impurities.^116^ Some methods to increase the lifetime of CaO are promising, such as hydration,^122^ doping with foreign ions,^123^ thermal pretreatment,^122^ nanomaterials,^124^ pelletization,^125^ and inert porous supports.^126^ However, none of these methods have solved this problem completely. Therefore, low-cost methods to improve the stability of CaO-based sorbents are still being explored.

### Alkaline thermal treatment with carbon mineralization

Metal hydroxides are attractive for *in situ* carbon capture and high purity H_2_ production during biomass gasification, and the process is called ATT.^127^ The most common hydroxide for ATT is sodium hydroxide, and the reaction between cellulose (a representative of biomass) and NaOH can be expressed as Reaction (3).

(Equation 3) C_6_H_10_O_5_ + 12NaOH + H_2_O → 6Na_2_CO_3_ + 12H_2_

After the biomass conversion process, NaOH could be regenerated by industrial wastes (e.g., steel slag and concrete waste) with CaO or Ca(OH)_2_ based on Reaction (4).

(Equation 4) Na_2_CO_3_ + Ca(OH)_2_ → CaCO_3_ + 2NaOH

The produced stable carbonate (CaCO_3_) is a type of mineral for permanent CO_2_ storage,^128^ and thus the whole process is called ATT with carbon mineralization, as shown in Figure 4. The main attractive aspect of ATT-CM is that the produced carbon minerals are ready for storage, which reduces the cost of impurity removal and compress of concentrated CO_2_ from other aforementioned technologies.^5^

The most common method of reacting NaOH with biomass is in the aqueous phase under subcritical or supercritical conditions. It was found that the H_2_ yield from cellulose could be increased by an order of magnitude when 1 M NaOH solution was used instead of water at 440°C and 35 Mpa. This was coupled with a substantial increase in organic carbon content through the carbonation of CO_2_, which in turn promoted the WGS reaction.^129^^,^^130^ Of all investigated literature, the inclusion of NaOH always has the effect of reduced CO and CO_2_ production coupled with enhanced H_2_ production.^129^^,^^130^^,^^131^^,^^132^^,^^133^

However, supercritical water gasification requires high pressure and specialized reactors, which led researchers to investigate the effects of alkali materials at ambient conditions in non-aqueous media. Ishida et al. have reported that H_2_ without CO_x_ could be produced from carbon, water, and group I hydroxides at ambient pressure and 600°C.^134^ They also showed that this reaction was possible with cellulose and indicated that high purity H_2_ could be obtained with suppressed CO and CO_2_ at mild temperature of 300°C.^135^ Stonor et al. compared different hydroxides and also found that group I alkali metals were highly effective at promoting H_2_ and suppressing carbonaceous side products.^136^ In a recent study, Zhou and Park investigated the ATT of real biomass feedstock (wheat straw) with NaOH at 500°C, and found negligible CO and very low CO_2_ (0.3 vol.%) were produced with a high purity of H_2_ (86.0 vol.%).^137^

One of the drawbacks of this technology is the high energy input during the regeneration of NaOH. Another concern of this process is the high corrosivity of NaOH. Therefore, Mg(OH)_2_ has also been investigated due to the better availability, lower cost, and lower corrosivity. Since Mg(OH)_2_ is available from minerals, regeneration is not needed. Mg(OH)_2_ can enhance the WGS through *in situ* mineral carbonation.^138^ However, Mg(OH)_2_ was found to be ineffective at promoting H_2_ production and mitigating CO_2_ release in the ATT reactions.^136^ Another challenge of Mg(OH)_2_ is that it decomposes at 350°C, which limits the reaction temperature at elevated temperatures.

## Biomass carbonization technologies

### Pyrolysis

**Figure 5:**
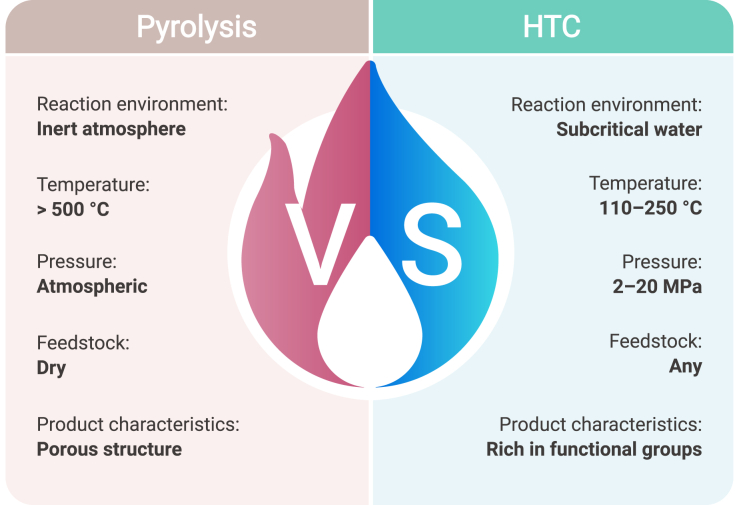
Comparison of biomass carbonization technologies, pyrolysis, and hydrothermal carbonization, in terms of reaction environment, temperature, pressure, feedstock, and product characteristics

Biomass pyrolysis is a process that converts biomass feedstocks into gaseous, liquid, and solid products by thermal decomposition (Figure 5).^139^ The process is usually carried out in an anoxic or anaerobic environment to avoid combustion of the biomass, and the products of pyrolysis mainly include bio-oil, syngas, and biochar.^140^^,^^141^ Bio-oil is a complex mixture containing a variety of organic compounds such as alcohols, ketones, and phenols, which can be used as fuel or further refined into chemicals.^142^^,^^143^ Syngas consists mainly of CO, H_2_, and small amounts of other gases such as CH_4_, and can be used to generate electricity or as a feedstock for the chemical industry.^144^ Biochar is a solid product consisting mainly of carbon and small amounts of minerals, which can be used as a carbon material for subsequent use.^145^^,^^146^ Given that the term “biochar” can refer broadly to all carbon materials derived from the thermochemical conversion of biomass, this section specifically uses biochar to refer to the solid-phase product generated through pyrolysis in order to clearly distinguish it from “hydrochar,” which is produced via hydrothermal carbonization.

It is worth noting that, among the three-phase products, the conversion of biomass to biochar has potential in terms of carbon emissions. This is due to the inherent carbon neutrality of biomass, and immobilization of biomass in a stable solid material can be equivalent to the capture and fixation of atmospheric CO_2_ of the same carbon content.^22^ However, pyrolysis of biomass does not always mean that a large share of biochar is produced, and the setting of the pyrolysis parameters in this process is crucial.^147^^,^^148^^,^^149^ In general, pyrolysis of biomass can be categorized as slow pyrolysis, fast pyrolysis, and flash pyrolysis. Fast pyrolysis and flash pyrolysis are usually carried out at high heating rates (100°C–1,000°C min^−1^) and high temperatures (500°C–1,200°C), with short reaction times (<10 s), and the main products are bio-oil or gas.^139^^,^^141^ Slow pyrolysis is characterized by lower heating rates (1°C–30°C min^−1^) and longer residence times (>10 min), which are more conducive to the formation of solid-phase biochar products.^146^^,^^150^

The slow pyrolysis process involves the gradual decomposition of biomass under slow heating and long reaction times, ultimately producing biochar. At room temperature up to 200°C, it is mainly the drying stage in which free and bound water in the biomass is evaporated and the volatile fraction removal stage in which some hydroperoxides, –COOH, and –CO groups are formed initially.^150^^,^^151^ When the temperature increases to 200°C–500°C, the cellulose, hemicellulose, and lignin in biomass decompose in large quantities, and various types of gas products and liquid products are eliminated, leaving a more stable biochar structure.^152^ When the temperature is above 500°C, a more stable carbon structure is generated, defining the main characteristics of biochar.^139^

The production of biochar through pyrolysis is a viable process because it is very simple and requires only one step and does not involve a complex process procedure. Generally, a fixed-bed reactor is commonly used for slow pyrolysis to produce biochar.^153^^,^^154^ The biomass is fixed in the bed of the reactor and a heat source is passed through the bed to simply heat the biomass. It should be noted that fixed bed is typically used in laboratories and is not suitable for large-scale production. Rotary kiln and belt reactor are more suitable for large-scale production of pyrolyzed biochar.^155^^,^^156^^,^^157^ Other types of reactors, such as fluidized bed reactors, are more suitable for fast pyrolysis processes due to their shorter reaction time, which is also not favorable for biochar formation. Biochar is suitable for a wide range of application scenarios in the energy and environment fields, especially in soil amendment. The porous structure of biochar allows it to improve the water retention, nutrient retention capacity, and aeration of soils, while increasing soil fertility through the promotion of microbial activity.^20^^,^^158^^,^^159^ Different from biomass, which is relatively easily decomposed by microorganisms in the soil, biochar derived from biomass exhibits stability several orders of magnitude higher,^20^^,^^160^ allowing it to serve as a long-term carbon sink in the soil. The high stability of biochar not only enables it to serve as an effective carbon storage carrier, but also makes it possible to provide a sustained impact on soil.

### Hydrothermal carbonization

Hydrothermal carbonization (HTC) of biomass is a technology that converts biomass to carbon material in a water environment at high temperature and pressure (Figure 5).^161^^,^^162^^,^^163^ Unlike conventional pyrolytic carbonization methods, HTC takes place in an aqueous environment and therefore does not require drying of the biomass and is capable of handling feedstocks with high moisture content. It is generally accepted that the HTC process typically takes place at temperatures in the range of 110°C–250°C, reaction pressures between 2 and 20 MPa, and reaction times ranging from a few hours to a few days, which are typically gentler reaction conditions than other hydrothermal reactions such as hydrothermal liquefaction and hydrothermal gasification.^164^^,^^165^^,^^166^

The HTC reaction can be divided into three stages, hydrolysis reaction, dehydration reaction, and repolymerization reaction.^167^^,^^168^^,^^169^ During the reaction, cellulose, hemicellulose, and lignin in the biomass undergo hydrolysis, generating small molecules of organic acids, sugars, and other soluble organic matter. The generated small-molecule organic matter further undergoes a dehydration reaction to generate carbon skeleton structure and release water simultaneously. The dehydrated organic matter then forms carbon materials with aromatic structures, called hydrochar, through reorganization and polymerization reactions. In recent years, there have also been some new research advances in developing new HTC methods and proposing reaction mechanisms for the direct conversion of biomass to hydrochar.^170^^,^^171^ They are usually differentiated by primary and secondary hydrochar.^163^^,^^172^ For primary hydrochar, a long reaction time is usually necessary because it promotes the conversion of small molecules from the liquid phase to the solid phase, thus increasing the yield of hydrochar. In the case of secondary hydrochar, the conversion of the solid phase to the solid phase can reach the equilibrium of the reaction quickly at the appropriate temperature and pressure.

Compared with the carbonization of biomass through pyrolysis, HTC does not require pre-drying and can handle biomass feedstocks with high moisture content.^173^^,^^174^ This means that HTC is able to fix carbon from a wider range of biomass, i.e., not only dry biomass such as wood, straw, and coconut shells, but also a wide range of agricultural waste, food waste, and sludge.^173^^,^^175^^,^^176^ In terms of reaction conditions, HTC has a lower reaction temperature and is sometimes considered to be more energy efficient than pyrolysis.^167^ However, considering the high latent heat of water and the high reaction pressure required for HTC, this is usually controversial and has not yet reached a good consensus. Moreover, there are some differences in the structural properties of hydrochar and pyrolytic biochar due to different reaction mechanisms. For example, pyrolytic biochars usually have high porosity and surface area, while hydrochars are rich in oxygen groups on the surface, such as hydroxyl, carboxyl, and carbonyl groups, which can affect their subsequent applications after carbon fixation.^159^^,^^177^^,^^178^

HTC technology, with its unique advantages in treating biomass with high water content, has achieved some initial industrial applications. However, reactor design for further process scale-up is a necessary problem to be solved due to the high pressure involved in the reactor. Concomitantly, the HTC process requires high-temperature and high-pressure conditions, with high equipment cost and energy consumption, so how to reduce the production cost and improve the economy is the key to achieve large-scale industrialization. Besides, HTC also produces process wastewater from liquid-phase products, and the utilization rate of this wastewater is still low.^179^^,^^180^^,^^181^^,^^182^ The need for reliable and feasible ways to utilize process wastewater will also be the key to the utilization of this technology.

## Comparison of carbon-negative emission technologies

### CO_2_ parameters

**Figure 6:**
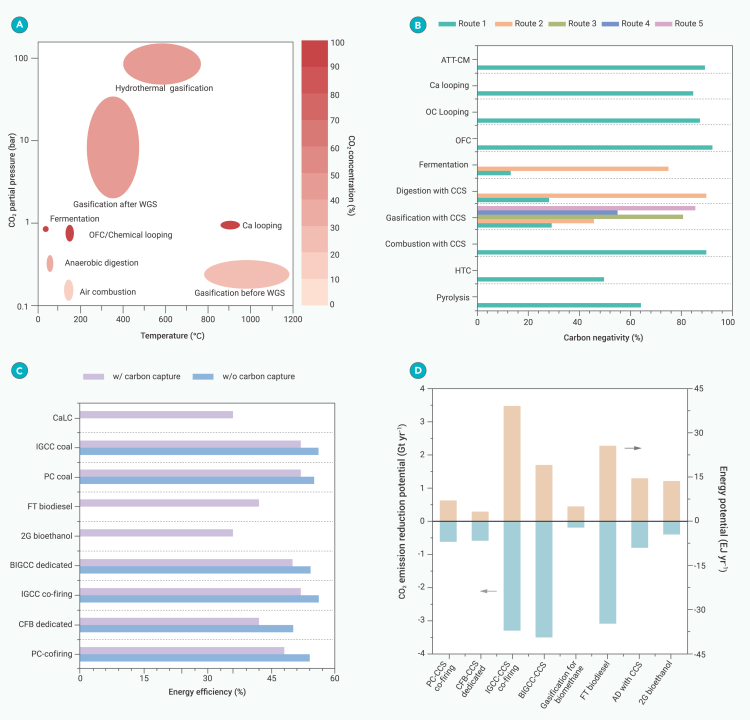
Comparison of different carbon-negative emission technologies enabled by biomass (A) CO_2_ status from different bio-energy conversion technologies (combustion,^183^ gasification,^65^ hydrothermal gasification,^167^^,^^184^ digestion,^76^^,^^185^ fermentation,^38^ OFC,^91^^,^^186^ chemical looping,^96^^,^^99^^,^^100^^,^^101^^,^^102^ and Ca looping^120^^,^^187^; average data are used for CO_2_ concentration). (B) Carbon negativity of different carbon-negative emission technologies enabled by biomass, including pyrolysis,^188^ HTC,^189^ combustion,^38^ gasification with CCS (based on Figure 3), digestion,^190^ fermentation,^38^ OFC,^91^ chemical looping,^96^^,^^99^^,^^100^^,^^101^^,^^102^^,^^111^ Ca looping,^120^ and ATT-CM.^137^ For gasification with CCS, routes 1, 2, 3, 4, and 5 represent IGCC without PCC, FT/methanol, hydrogen, synthetic natural gas, and IGCC with PCC, respectively. For digestion with CCS, routes 1 and 2 represent biomethane utilization without and with CCS, respectively. For fermentation, routes 1 and 2 represent lignin utilization without and with CCS, respectively. (C) Biomass conversion efficiency (based on lower heating value) with or without carbon capture from a 2050 perspective (PC [pulverized coal] post-combustion capture is not considered for IGCC).^38^ (D) CO_2_ emission reduction potential and energy potential of different BECCS routes from a 2050 perspective (the share of fossil fuels is also included in the energy potential).^38^^,^^190^

To compare the CO_2_ stream from different biomass utilization methods, the CO_2_ temperature, partial pressure, and concentration from different pathways are summarized (Figure 6A). For combustion, gasification, and digestion, the CO_2_ concentration is low, and thus additional CO_2_ capture processes are needed. For integrated BECCS technologies, such as biomass fermentation, OFC, chemical looping, and Ca looping, the CO_2_ is already concentrated, and additional CO_2_ capture process may not be needed. The temperature and CO_2_ partial pressure are also essential for the capture technologies. Capture technologies that operated at high temperatures and high pressures (such as CaO) show advantages for the CO_2_ capture from biomass gasification, where the energy of heating and pressure could be saved. The CO_2_ released from Ca looping is at high temperatures of 900°C–950°C, and thus a heat exchanger could be designed to pre-heat the input feedstocks and cool down the CO_2_ for transportation.

### Carbon negativity

To describe the carbon-negative potential of a BECCS technology, carbon negativity is proposed herein, which is defined as the total carbon in biomass divided by the carbon that is captured and stored (Figure 6B). Biomass combustion, OFC, chemical looping, and Ca looping have a relatively high carbon negativity of ∼90%. The carbon negativity of IGCC without PCC is only ∼50%, while this number could be as high as ∼90% when the PCC is applied in the IGCC process. For biomass gasification with FT, around 51%–54% of the carbon is captured, and 23%–32% of the carbon remains in the FT biodiesel.^191^^,^^192^ The remaining carbon will be emitted from the combustion of tail gas. For biomethane product from the AD process, the carbon negativity value is less than 30%, while the carbon-negative potential should increase significantly if the carbon is captured during the utilization of biomethane. For biomass fermentation, the carbon negativity is very low, since a large amount of carbon is in the form of unreacted lignin.^193^ In contrast, the carbon negativity of carbonization techniques, including pyrolysis and hydrothermal carbonization, is moderate, approximately 50%–70%.

### Energy efficiency

Energy efficiency is very important for a biomass utilization method. The energy efficiencies (based on lower heating value) of different BECCS technologies from a 2050 perspective are estimated (Figure 6C). With the CCS, the efficiencies decrease by 4%–8%. Co-fired IGCC-CCS presents the highest efficiency (52%), which is similar to the IGCC-CCS of coal. The 2G bioethanol (36%) and FT biodiesel (42%) have relatively low conversion efficiencies, while the liquid products are important substitutes for transportation fuels.

### CO_2_ emission reduction and energy potential

The International Energy Agency Greenhouse Gas R&D Program (IEAGHG)^38^^,^^190^ reported the potential of different types of BECCS technologies in 2050 (Figure 6D). For heat and electricity production, the largest potential was found to be gasification-based routes, including co-fired IGCC-CCS and BIGCC-CCS. According to Klein et al., BIGCC with CCS could be the main long-term bio-energy conversion method, representing 33% of global carbon mitigation by 2100.^194^ It should be noted that co-fired systems have larger potential than dedicated systems. Gough and Upham pointed out that more than 20% of biomass co-firing is needed for BECCS systems.^32^ The Commission of European Commodities also suggested that biomass co-firing with CCS is the most promising technology for electricity from renewable energy with low-risk and low-cost CO_2_ mitigation.^195^ For biofuels production, the largest potential was found to be FT biodiesel at 26 EJ per year, which could remove 3 Gt CO_2_ per year in 2050. As shown in Figure 6D, electricity production is preferred to biofuel production. This is similar to the estimation of Luckow et al., which shows that BECCS electricity will be the dominant bio-energy utilization method after 2050 according to 400 or 450 ppmv constraints.^196^

### Economic viability and commercialization status

Although carbon-negative emission technologies enabled by biomass have significant environmental and economic potential in the long term, their current economics remain challenging.^197^ The main reasons for the high price of biomass-based carbon-negative technologies include the complexity and high cost of CCS technology, the high cost due to the decentralization of biomass collection, treatment, and transportation, and the relatively low efficiency of energy conversion and the large initial infrastructure investment.^198^^,^^199^ Furthermore, the overall technologies are still in their early stages, with economies of scale yet to be achieved, leading to high maintenance and operational costs. Stringent policy and regulatory compliance requirements also add to project costs.^197^^,^^200^ Together, these factors make the current economics challenging for these technologies, despite their great potential to combat climate change.

**Figure 7:**
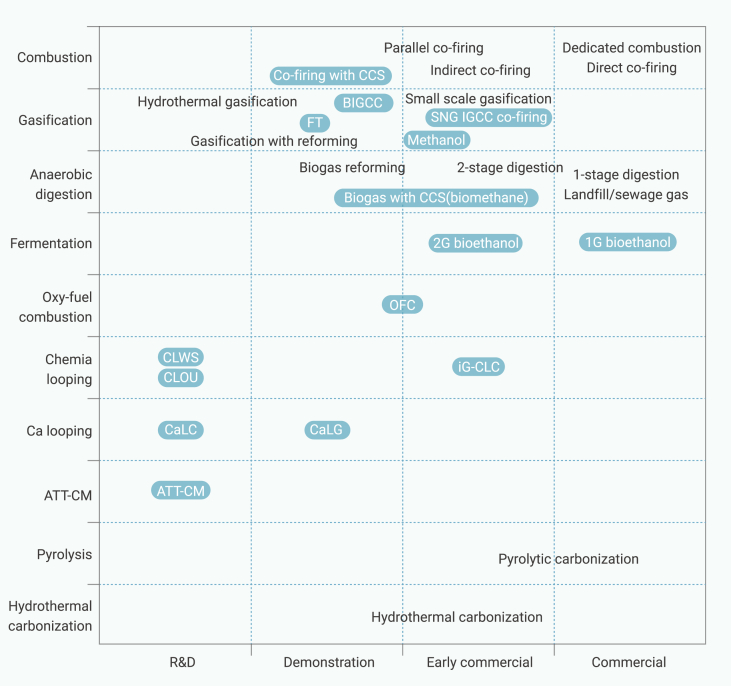
Current development status of carbon-negative emission technologies enabled by biomass Black text: biomass conversion technologies without CCS; white text: biomass conversion technologies with CCS (BECCS technologies).^62^^,^^201^^,^^202^

Therefore, among different types of biomass utilization technologies, only combustion (dedicated or directly co-fired), one-stage digestion, landfill/sewage gas, and 1G bioethanol production are commercialized (Figure 7). With 1G bioethanol a mature BECCS technology, combustion with CCS and biogas production with CCS (biomethane production) provided early opportunities.^32^^,^^203^ The carbonization technologies of pyrolytic carbonization and hydrothermal carbonization are in the stage of early commercialization, but have not yet achieved full commercial profitability. Biomass gasification with subsequent carbon capture is promising, due to the separation of CO_2_ from syngas, which could increase the partial pressure of H_2_ and CO for downstream conversions. Continuous progress is obtained every year for 2G bioethanol, which promises a positive future for the commercialization.^86^ OFC and iG-CLC may provide an early possibility for novel integrated BECCS technologies.^204^^,^^205^ Other integrated BECCS technologies are still waiting for further technical breakthroughs.

The first BECCS project, Russell EOR Research Project, was operated in Kansas, USA. The main purpose of the project was to evaluate the possibility of EOR with CO_2_. From December 2, 2003, to June 21, 2005, the project injected 7,700 tons of CO_2_ from biogenic source.^33^ Currently, there are six BECCS projects in operation that capture 2.16 Mt CO_2_ every year (Figure S2; Table S3), and all of them are 1G bioethanol plants combined with EOR. Other technologies, such as biogas with CCS, gasification with CCS, co-fired IGCC-CCS, and combustion with CCS are still under construction or evaluation.

Indeed, several countries and regions have promoted BECCS technology through policy incentives. In the US, the Inflation Reduction Act strongly supports CCS technology through 45Q tax credits, and the federal government has initiated a program to enter into offtake agreements with carbon dioxide removal suppliers, including those utilizing BECCS technologies, making BECCS programs more economically attractive. The European Union’s Green New Deal funds BECCS technologies through the Emissions Trading System and R&D programs such as Horizon Europe, especially in the Nordic countries such as Sweden, Finland, and Denmark, where the widespread use of biomass energy provides a sustainable resource base for BECCS. For example, the Danish Energy Agency has awarded contracts to three BECCS projects in 2024 under the fund for negative CO_2_ emissions (NECCS fund).^206^ Moreover, Japan views BECCS as part of a hydrogen economy, using biomass to produce “carbon-negative hydrogen” and supporting technology development through energy innovation subsidies and tax incentives. In China, the development of CCS technology is explicitly mentioned in its 14th Five-Year Plan, and BECCS is likely to be a key area for future regional pilot and demonstration programs. These policy incentives have been an important driver for the continued development of BECCS technologies in global carbon-neutral and carbon-negative strategies.

## Challenges and outlook

Though carbon-negative emission technologies enabled by biomass is a low-hanging fruit among the carbon-negative technologies, there are many challenges that require further research.

### Effect of impurities on CO_2_ capture

For post-combustion capture of a coal-fired power plant, the impurities are mainly SO_x_, NO_x_, and PMs.^207^ However, depending on different BECCS technologies, there are various impurities in the gas stream. For biomass post-combustion capture, the fly ash containing alkali metal salts is one of the main impurities.^51^^,^^208^ For biomass AD, NH_3_ and H_2_S are generated due to the protein in the biomass.^76^ HCl and even dioxins are usually presented in the flue gas of waste incineration.^209^^,^^210^ Therefore, more studies are needed to explore the influence of impurities on the CO_2_ capture for BECCS technologies.

### Co-capture and co-storage of CO_2_ and impurities

Co-capture of H_2_S and CO_2_ has the advantage of system simplicity and cost reduction, while it is critical for H_2_S and CO_2_ to be regenerated separately.^211^ The co-capture of CO_2_ and SO_2_ from combustion by CaO has also been proposed, while the regeneration of CaO from CaSO_4_ is a challenge due to poor kinetics.^212^ During biomass combustion or OFC, the co-storage of CO_2_ together with SO_x_, NO_x_, non-condensable gases, and water is proposed,^213^ which should reduce the capital and operating costs of the power plant. However, the presence of corrosive gases and water requires more resistant materials for compressors and pipelines.^91^ In addition, since CO_2_ is most likely transported in a supercritical phase in the pipeline, inert gases may lead to the two-phase flow in the pipeline and lead to a considerable increase of flow resistance. The co-capture and co-storage of CO_2_ and impurities are still very challenging.

### Application of new biomass feedstocks

Currently, there are some new types of biomass feedstocks especially for energy use, including energy crops and algae, which are promising for large-scale carbon-negative applications since they can be grown on poor/degraded soils or even waste water/seawater.^214^^,^^215^ The composition of these new feedstocks might be different from traditional biomass. For example, algae have no lignin and low hemicellulose content, which could lead to high hydrolysis efficiency and high fermentation yields.^216^ Therefore, the application of these new biomass types needs to be further explored.

### The flexibility of bio-energy utilization systems

Due to the seasonality of plant growth, it is not easy to have a steady single biomass source, which means that bio-energy utilization may face challenges in handling the variety of biomass feedstocks. Therefore, the flexibility of bio-energy utilization system is very important, since different biomass feedstocks may present distinct characteristics. Some technologies already present advantages due to the flexibility, such as CFB combustion which can adapt to different types of fuels including lignocellulosic biomass, municipal solid waste, plastics, and even sewage sludge,^60^ while more technologies with better flexible potential are still being explored.

### Distributed BECCS technology

Compared with fossil fuel energy, biomass is widely distributed on Earth with low energy density. Therefore, the collection and transportation of biomass can be quite costly, which means that the implementation of bio-energy is thus expected to be small scale or even mobile. However, the efficiencies of most of the industrial applications decrease with the decrease of unit capacity. For example, it is estimated that the efficiency of a 100 MW biomass CLC power plant is 38%, while the efficiency of a 15 MW biomass CLC power plant is only 32%.^217^ Therefore, the Organic Rankine Cycle is proposed for small power plants, which may have higher efficiency with working medium with low temperature and pressure.^218^ The limited CO_2_ storage sites also raise the issue of costs associated with cost of CO_2_ transportation from the biomass utilization facilities to the CO_2_ storage sites.

### Integrated utilization of BECCS technologies

The aforementioned BECCS technologies can be combined to create a greater carbon-negative effect. For example, 2G bioethanol has 60%–65% carbon stream in the form of lignin and residue, which is refractory during the biochemical process.^38^ This lignin can be used in a thermochemical BECCS pathway, such as combustion or gasification with CCS, which could increase the carbon negativity of the entire process considerably.

### Practical carbon-negative emission potential

When assessing carbon negativity from biomass conversion technologies or biomass carbonization technologies in practice, apart from the conversion technology itself, the front-end processes, such as biomass acquisition, energy demand during pre-processing, and transportation activities, are key aspects that should not be neglected, and are particularly important in life cycle assessment (LCA). These preliminary stages not only involve direct consumption of energy, but may also be accompanied by significant greenhouse gas emissions, which can have a substantial impact on the final assessment results. Therefore, when considering the practical situation and constructing the LCA model of a biomass conversion pathway, it is necessary to systematically incorporate these links to ensure that the assessment of carbon emission reduction benefits is comprehensive and scientific, and to avoid bias or overestimation due to improper boundary setting.

### Future fossil-free energy systems

Future fossil-free energy systems will be in a diversified and synergistic energy ecosystem, in which bio-energy and its related carbon-negative technologies will play a key role. It will not only serve as a stable source of energy, but will also have a positive climate impact by capturing and sequestering CO_2_. By integrating with renewable energy sources, promoting hydrogen production from biomass, and expanding the biomass valorization chain, bio-energy is expected to become one of the key pillars in future fossil-free energy systems. With the dual impetus of policy and technology, carbon-negative technologies enabled by biomass will provide important support for the realization of global climate change goals.

### Economic challenges

While carbon-negative emission technologies enabled by biomass are strategically important, challenges remain in its economics. Especially in scenarios where biomass resources are constrained, the system cost may increase significantly.^219^ Therefore, technological advances, policy incentives, and carbon pricing are needed to reduce the cost and enhance the economic viability of **c**arbon-negative emission technologies enabled by biomass in the future. Improvements in carbon pricing and carbon markets can help promote these technologies and make them more economically attractive. By establishing clear CCS policies, providing financial incentives and subsidies, and promoting regulations for sustainable biomass production, governments can provide guarantees for the widespread adoption of carbon-negative emission technologies enabled by biomass technologies.

The CCS has been widely discussed for fossil fuel utilization, but this is also suitable for bio-energy utilization, which will develop this process from “carbon neutral” to “carbon negative.” When a CO_2_ pricing mechanism is introduced, more opportunity will be given to carbon-negative emission technologies enabled by biomass. This paper reviews various processes and biomass conversion routes with the same common goal: to produce heat, electricity, fuels, carbon materials, or chemicals with negative CO_2_ release. Carbonization technology enables the direct fixation and storage of carbon in biomass. Other technologies require the capture of CO_2_ at some point of the processes. For biomass combustion, gasification, or AD, various types of CCS technologies could be applied, which means that the optimal one should be selected based on the CO_2_ status such as temperature, pressure, concentration, and impurities. Some integrated BECCS technologies, including fermentation, OFC, chemical looping, calcium looping, and ATT with carbon mineralization also show significant potentials since the additional CCS process is not necessary. While most of the carbon-negative emission technologies enabled by biomass are still in the early development stage, more studies are required to break the technical bottleneck and enhance the economic feasibility.

## Funding and Acknowledgments

This work was supported by the 10.13039/501100001809National Natural Science Foundation of China (52276202), 10.13039/501100012166National Key R&D Program of China (2023YFC3905701), Huaneng Group Science and Technology Research Project (KTHT-U23GCZH01, KTHT-U22YYJC12), Tsinghua-Jiangyin Innovation Special Fund (TJISF), Tsinghua-Toyota Joint Research Fund, and 10.13039/501100011308State Key Laboratory of Chemical Engineering (SKL-ChE-22A01). Prof. J. Yan would like to acknowledge the 10.13039/501100004377Hong Kong Polytechnic University for the financial support (P0043885 - Flexibility of Urban Energy Systems [FUES] and P0047700 - International Centre of Urban Energy Nexus [UEX]).

## Declaration of interests

The authors declare no competing interests.

## Supplemental information

Document S1. Figures S1 and S2 and Tables S1–S3

Document S2. Article plus supplemental information
