# Supplementary material for: Enhancing carbon-negative emission technologies through biomass integration
Source: Innovation (Camb). 2025 Aug 8;6(12):101079. doi: 10.1016/j.xinn.2025.101079 (PMC12859671; doi:10.1016/j.xinn.2025.101079)
Supplement: Document S1. Figures S1 and S2 and Tables S1–S3 [file mmc1.pdf]

The Innovation, Volume *6*

Supplemental Information

Enhancing carbon-negative emission technologies through biomass integration

Shijie Yu, Qinghai Li, Yanguo Zhang, Jinyue Yan, and Hui Zhou

Supplementary Information

Enhancing Carbon-Negative Emission Technologies Through Biomass Integration

Shijie Yu[1,2], Qinghai Li[1,3], Yanguo Zhang[1]*, Jinyue Yan[4,5]*, Hui Zhou[1,3]*

[1]*Key Laboratory for Thermal Science and Power Engineering of Ministry of Education, Beijing Key Laboratory of CO_2 Utilization and Reduction Technology, Department of Energy and Power Engineering, Tsinghua University, Beijing 100084, China*

[2]*Department of Chemical and Biomolecular Engineering, National University of Singapore, Singapore 117585, Singapore*

[3]*Shanxi Research Institute for Clean Energy, Tsinghua University, Shanxi, Taiyuan 030000, China*

[4]*Department of Building Environment and Energy Engineering, The Hong Kong Polytechnic University, Hong Kong SAR 999077, China*

[5]*International Centre of Urban Energy Nexus, The Hong Kong Polytechnic University, Hong Kong SAR 999077, China*

**Correspondence: zhangyg@tsinghua.edu.cn (Y.Z.), j-jerry.yan@polyu.edu.hk (J.Y.), huizhou@tsinghua.edu.cn (H.Z.)*

Table S1. Examples of carbon capture technologies for different situations

**Bio-energy utilization method**	**Combustion**[1–5]	**Gasification**[1–3,5,6]	**Biogas**[1–3,5,7]
CO_2 concentration	~15%	10-40% (before WGS)<br>20-50% (after WGS)<br>20-40% (hydrothermal)	25-45%
Impurities	Water, O_2, NO_x, SO_x, HCl, HF, and PMs	Water, CO, H_2S, NH_3, HCl, Tar, and PMs	Water, H_2S, and NH_3
Temperature (°C)	120-150	750-1150 (before WGS)<br>200-450 (after WGS)<br>200-600 (hydrothermal)	25-70
Chemical solvents	**Amine-based solvents**<br>**Ammonia**<br>**Amino acid salts**<br>Carbonates<br>Enzymatic<br>Functionalized ionic liquids	**Amine-based solvents**<br>Carbonates<br>Hybrid physical/chemical solvent<br>Enzymatic	**Amine-based solvents**<br>Carbonates<br>Hybrid physical/chemical solvent<br>Enzymatic
Physical solvents	Ionic liquids	**Glycol**<br>**Methanol**<br>Ionic liquids<br>Hydrates	**Glycol**<br>**Methanol**<br>**High-pressure water wash**
Chemical sorbents	CaO-based sorbents<br>Carbonates<br>Mineral carbonation<br>MOFs<br>Solid amine	CaO-based sorbents<br>Carbonates<br>Hydrotalcites<br>Li_2ZrO_3<br>MOFs	
Physical solvents	Zeolites<br>Activated carbon<br>Carbon based sorbents<br>MOFs	Zeolites<br>Activated carbon<br>Alumina silicates<br>MOFs	Zeolites<br>Activated carbon<br>Alumina silicates
Membranes	Polymeric<br>Hollow fiber membrane contractor<br>Ceramic<br>Facilitated transport membranes	Polymeric<br>Hollow fiber membrane contractor	**Polymeric**<br>Hollow fiber membrane contractor<br>Ceramic<br>Facilitated transport membranes

	Carbon<br>Supported ionic liquid membranes<br>Hybrid membrane/amine		Carbon<br>Hybrid membrane/amine
Cryogenic	Liquefaction<br>Hybrid processes	Liquefaction<br>Hybrid processes	Ryan-Holmes process

Processes shown in bold are currently preferred technologies that have been commercialized.

Table S2. Calculation of gas composition and carbon stream during syngas utilization

Routes/final product			IGCC	SNG	Syncrude/Methanol	H_2
Raw biomass	C stream (%)		100	100	100	100
Syngas[a]	Gas composition (%)	H_2	30	30	30	30
		CO	40	40	40	40
		CO_2	25	25	25	25
		CH_4	5	5	5	5
	C stream (%)	CO	51	51	51	51
		CO_2	32	32	32	32
		CH_4	6	6	6	6
		Total	90	90	90	90
Reforming process	Gas composition (%)	H_2	NA[b]	NA[c]	39	39
		CO			39	39
		CO_2			22	22
		CH_4			0	0
WSG process	HTC ratio		NA[b]	3	2	∞
	Gas composition (%)	H_2		43	46	56
		CO		14	23	0
		CO_2		39	31	44
		CH_4		4	0	0
	C stream (%)	CO		23	39	0
		CO_2		61	51	90
		CH_4		6	0	0
Carbon capture	C stream (%)		29	55	35	81
Final product	C stream (%)		57[d]	26	35	0

a. The gasification carbon conversion and syngas composition depends on many factors such as biomass type, furnace type, temperature, gasifying agent. Here, the gasification carbon conversion is supposed to by 90%, which means the other 10% in the form of biochar or tar. The syngas composition is averaged from results in Rauch R et al., 2014-3.

b. IGCC uses syngas directly without reforming and WSG.

c. The SNG production process do not need the process of reforming, since methane can be considered inert during the synthesis reaction [8].

d. It is flue gas here.

In this table, the carbon conversion efficiencies of all carbon capture processes and synthesis reactions are regarded to be 90%. The carbon conversion efficiency of IGCC combustion in gas turbine is regarded to be 100%.

Table S3. List of global BECCS projects [9–22].

Index	Project name/leadership	Location
a	Russell EOR Research Project	Russell, KS, USA
b	Sao Paulo Project	Sao Paulo State, Brazil
c	Husky Energy	Lashburn, SK, Canada
d	Bonanza Bioethanol	Garden City, KS, USA
e	Lantmännen Agroetanol	Norrköping, Sweden
f	CPER Artenay Project	Artenay and Toury, France
g	Arkalon Bioethanol	Liberal, KS, USA
h	RCI/OCAP/ROAD	Rotterdam, Netherlands
i	Illinois Industrial CCS	Decatur, IL, USA
j	Mikawa Power Plant	Omuta, Fukuoka, Japan
k	Klemetstrud	Oslo, Norway
l	Södra	Värö, Sweden
m	Norcem	Brevik, Norway
n	Drax Power Ltd.	Selby, UK
o	C.GEN North Killingholme Power Project	North Killingholme, UK
p	Biorecro/Energy and Environmental Research Center	ND, USA
q	Domsjö Fabriker	Domsjö Sweden
r	Skåne	Skåne, Sweden

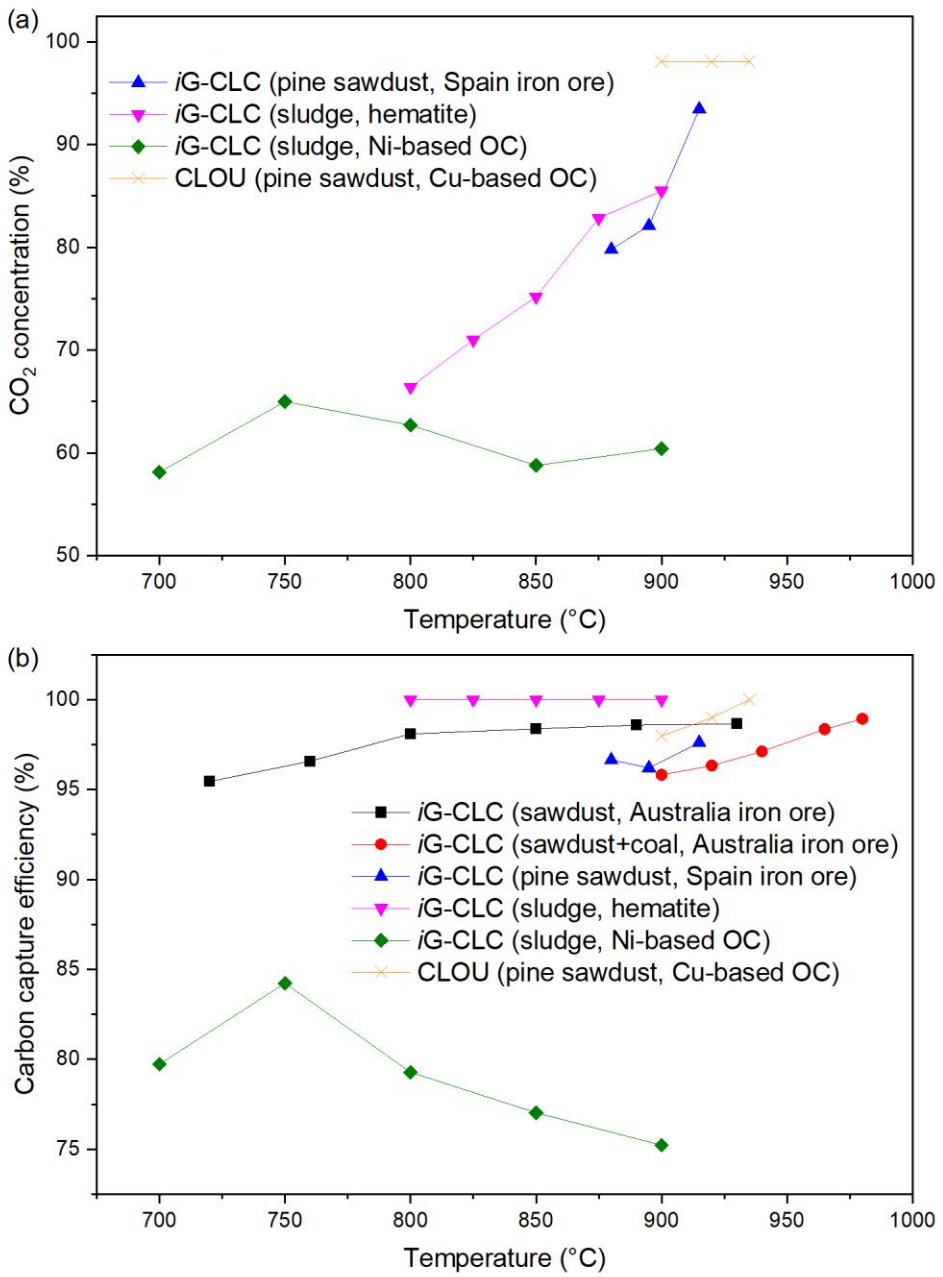

Figure S1. CO_2 concentration and carbon capture efficiency of different kinds of CLCs at different fuel reactor temperatures [23–27].

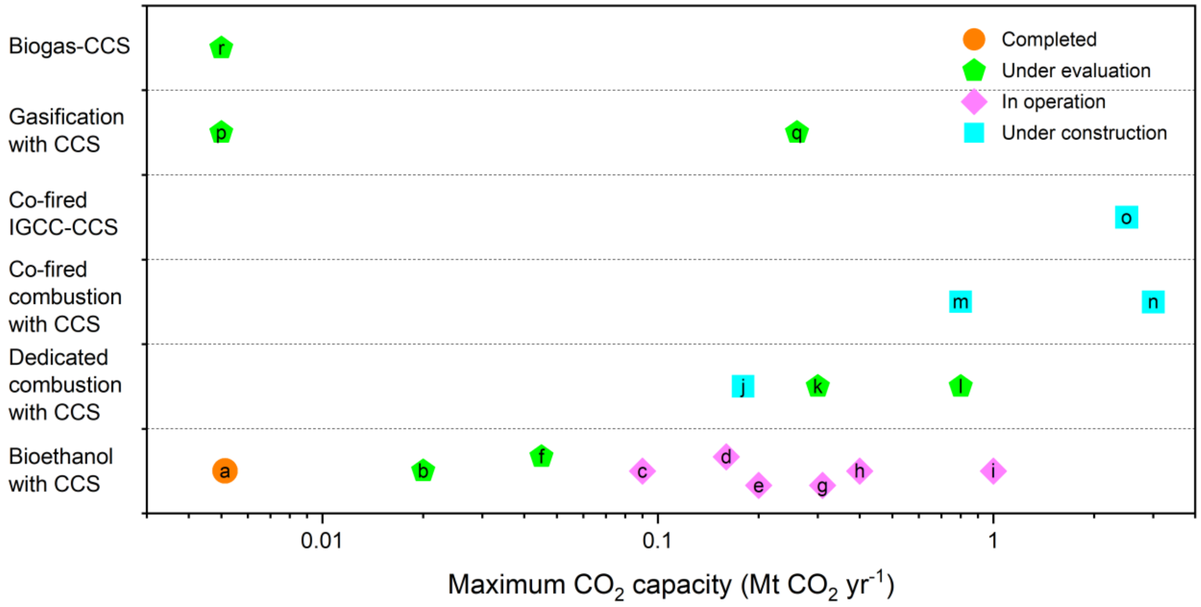

Figure S2. Global BECCS projects (for the co-fired projects, the CO_2 capacity includes the share of fossil fuels; waste-to-energy power plant is considered as dedicated combustion; see Table S3 for details of the name and location of the projects) [9–22,28–30].

References

1. Intergovernmental Panel on Climate Change. *Carbon dioxide capture and storage*. (Cambridge University Press, 2005).
2. Boot-Handford, M. E. *et al.* Carbon capture and storage update. *Energy Environ. Sci.* **7**, 130–189 (2014).
3. Global CCS Institute. *Available CO2 capture technology types*. (2016).
4. Moazzem, S., Rasul, M. G. & Khan, M. *A review on technologies for reducing CO2 emission from coal fired power plants*. (INTECH Open Access Publisher, 2012).
5. Pires, J. C. M., Martins, F. G., Alvim-Ferraz, M. C. M. & Simões, M. Recent developments on carbon capture and storage: An overview. *Chem. Eng. Res. Des.* **89**, 1446–1460 (2011).
6. Peterson, A. A. *et al.* Thermochemical biofuel production in hydrothermal media: A review of sub- and supercritical water technologies. *Energy Environ. Sci.* **1**, 32 (2008).
7. Ryckebosch, E., Drouillon, M. & Vervaeren, H. Techniques for transformation of biogas to biomethane. *Biomass Bioenergy* **35**, 1633–1645 (2011).
8. Rauch, R., Hrbek, J. & Hofbauer, H. Biomass gasification for synthesis gas production and applications of the syngas. *Wiley Interdiscip. Rev. Energy Environ.* **3**, 343–362 (2014).
9. Rotterdam, C. I. CO2 capture and storage in Rotterdam-A network approach. *Rotterdam Rotterdam Clim. Initiat.* (2011).
10. DiPietro, P., Balash, P. & Wallace, M. A note on sources of CO2 supply for enhanced-oil-recovery operations. *SPE Econ. Manag.* **4**, 69–74 (2012).
11. Gollakota, S. & McDonald, S. CO2 capture from ethanol production and storage into the Mt Simon Sandstone. *Greenh. Gases Sci. Technol.* **2**, 346–351 (2012).

12. Junginger, M., Goh, C. S. & Faaij, A. *International Bioenergy Trade: History, status & outlook on securing sustainable bioenergy supply, demand and markets*. vol. 52 (Springer Science & Business Media, 2013).
13. Finley, R. J. An overview of the Illinois Basin – Decatur Project. *Greenh. Gases Sci. Technol.* **4**, 571–579 (2014).
14. Bjerge, L.-M. & Brevik, P. CO2 Capture in the Cement Industry, Norcem CO2 Capture Project (Norway). *Energy Procedia* **63**, 6455–6463 (2014).
15. Jones, R. A. & McKaskle, R. W. Design and operation of compression system for one million tonne CO2 sequestration test. *Greenh. Gases Sci. Technol.* **4**, 617–625 (2014).
16. Mastop, E. A., Best-Waldhober, M. D., Hendriks, C. & Ramirez-Ramirez, A. Informed public opinions on CO2 mitigation options in the Netherlands: deliberating expert information and lay beliefs. *Policy Stud.* **2014**, 2013 (2015).
17. Global CCS Institute. *Large Scale CCS Projects*. (2015).
18. Global CCS Institute. *Notable Projects*. (2015).
19. Global CCS Institute. *White Rose CCS Project*. (2015).
20. NETL. *Recovery Act: CO2 Capture From Biofuels Production and Sequestration into the Mt. Simon Sandstone Reservoir*. (2015).
21. Kemper, J. Biomass and carbon dioxide capture and storage: A review. *Int. J. Greenh. Gas Control* **40**, 401–430 (2015).
22. Brevik, P. & Bjerge, L. M. *Carbon capture – a part of our zero vision*. (2017).
23. Gu, H., Shen, L., Xiao, J., Zhang, S. & Song, T. Chemical looping combustion of biomass/coal with natural iron ore as oxygen carrier in a continuous reactor. *Energy Fuels* **25**, 446–455 (2011).

24. Mendiara, T. *et al.* Biomass combustion in a CLC system using an iron ore as an oxygen carrier. *Int. J. Greenh. Gas Control* **19**, 322–330 (2013).

25. Mendiara, T. *et al.* Process Comparison for Biomass Combustion: In Situ Gasification-Chemical Looping Combustion (iG-CLC) versus Chemical Looping with Oxygen Uncoupling (CLOU). *Energy Technol.* **4**, 1130–1136 (2016).

26. Niu, X., Shen, L., Gu, H., Jiang, S. & Xiao, J. Characteristics of hematite and fly ash during chemical looping combustion of sewage sludge. *Chem. Eng. J.* **268**, 236–244 (2015).

27. Niu, X., Shen, L., Gu, H., Song, T. & Xiao, J. Sewage sludge combustion in a CLC process using nickel-based oxygen carrier. *Chem. Eng. J.* **260**, 631–641 (2015).

28. OGJ editors. Oxy, White Energy studying possible carbon-capture plant. *Oil Gas J.* (2018).

29. Construction Work Progresses at Large-Scale Carbon Capture Demonstration Facility : Articles/Topics : Thermal Power | TOSHIBA ENERGY SYSTEMS & SOLUTIONS CORPORATION. https://www.toshiba-energy.com/en/thermal/topics/ccs-1.htm (2019).

30. Bioenergy Carbon Capture and Storage (BECCS) Task Force. *Technical Summary of Bioenergy Carbon Capture and Storage (BECCS)*. (2018).
